# eIF4A/PDCD4 Pathway, a Factor for Doxorubicin Chemoresistance in a Triple-Negative Breast Cancer Cell Model

**DOI:** 10.3390/cells11244069

**Published:** 2022-12-15

**Authors:** Alina González-Ortiz, Angel Pulido-Capiz, César Y. Castañeda-Sánchez, Esmeralda Ibarra-López, Octavio Galindo-Hernández, Maritza Anahí Calderón-Fernández, Leslie Y. López-Cossio, Raul Díaz-Molina, Brenda Chimal-Vega, Nicolás Serafín-Higuera, Iván Córdova-Guerrero, Victor García-González

**Affiliations:** 1 Departamento de Bioquímica, Facultad de Medicina Mexicali, Universidad Autónoma de Baja California, Mexicali 21000, Mexico; 2 Laboratorio Multidisciplinario de Estudios Metabólicos y Cáncer, Universidad Autónoma de Baja California, Mexicali 21000, Mexico; 3 Laboratorio de Biología Molecular, Facultad de Medicina Mexicali, Universidad Autónoma de Baja California, Mexicali 21000, Mexico; 4 Facultad de Odontología Mexicali, Universidad Autónoma de Baja California, Mexicali 21000, Mexico; 5 Facultad de Ciencias Químicas e Ingeniería, Universidad Autónoma de Baja California, Tijuana 22424, Mexico

**Keywords:** breast cancer, chemoresistance, PDCD4, eIF4A, cryptotanshinone, proteostasis

## Abstract

Cells employ several adaptive mechanisms under conditions of accelerated cell division, such as the unfolded protein response (UPR). The UPR is composed of a tripartite signaling system that involves ATF6, PERK, and IRE1, which maintain protein homeostasis (proteostasis). However, deregulation of protein translation initiation could be associated with breast cancer (BC) chemoresistance. Specifically, eukaryotic initiation factor-4A (eIF4A) is involved in the unfolding of the secondary structures of several mRNAs at the 5′ untranslated region (5′-UTR), as well as in the regulation of targets involved in chemoresistance. Importantly, the tumor suppressor gene PDCD4 could modulate this process. This regulation might be disrupted in chemoresistant triple negative-BC (TNBC) cells. Therefore, we characterized the effect of doxorubicin (Dox), a commonly used anthracycline medication, on human breast carcinoma MDA-MB-231 cells. Here, we generated and characterized models of Dox chemoresistance, and chemoresistant cells exhibited lower Dox internalization levels followed by alteration of the IRE1 and PERK arms of the UPR and triggering of the antioxidant Nrf2 axis. Critically, chemoresistant cells exhibited PDCD4 downregulation, which coincided with a reduction in eIF4A interaction, suggesting a sophisticated regulation of protein translation. Likewise, Dox-induced chemoresistance was associated with alterations in cellular migration and invasion, which are key cancer hallmarks, coupled with changes in focal adhesion kinase (FAK) activation and secretion of matrix metalloproteinase-9 (MMP-9). Moreover, eIF4A knockdown via siRNA and its overexpression in chemoresistant cells suggested that eIF4A regulates FAK. Pro-atherogenic low-density lipoproteins (LDL) promoted cellular invasion in parental and chemoresistant cells in an MMP-9-dependent manner. Moreover, Dox only inhibited parental cell invasion. Significantly, chemoresistance was modulated by cryptotanshinone (Cry), a natural terpene purified from the roots of *Salvia brandegeei*. Cry and Dox co-exposure induced chemosensitization, connected with the Cry effect on eIF4A interaction. We further demonstrated the Cry binding capability on eIF4A and in silico assays suggest Cry inhibition on the RNA-processing domain. Therefore, strategic disruption of protein translation initiation is a druggable pathway by natural compounds during chemoresistance in TNBC. However, plasmatic LDL levels should be closely monitored throughout treatment.

## 1. Introduction

Triple-negative breast cancer (TNBC) accounts for 15% to 20% of annual breast cancer (BC) diagnoses [1]. The name of this condition refers to the fact that the BC cells fail to express estrogen/progesterone receptors as well as human epidermal growth factor receptor 2 (HER2) [2,3,4], and this type of cancer is diagnosed based on immunohistochemical criteria [2]. TNBC has a poor prognosis due to the lack of specific and effective treatments. TNBC is a highly aggressive cancer with poor clinical outcomes due to its high degree of recurrence and distant metastasis. Therefore, the survival rates of TNBC patients are far lower than those diagnosed with other BC subtypes. Histologically, TNBC is a high-grade invasive carcinoma, and is molecularly classified as a basal phenotype. TNBC is characterized by a low sensitivity to the targeted receptor and endocrine therapies, as well as a high rate of chemoresistance [2,4,5]. 

The unfolded protein response (UPR) is composed of a tripartite signaling system that regulates gene expression in response to cellular stress and maintains protein homeostasis (proteostasis) through the regulation of protein translation [6]. Accumulation of unfolded proteins in the endoplasmic reticulum (ER) lumen triggers the activation of the three UPR transducers, ATF6, PERK, and IRE1, which promote mRNA degradation and translation of chaperones through the activation of the transcription factors ATF6α, CHOP, and XBP1s, respectively [7]. Critically, these mechanisms are altered in BC cells [8]. Moreover, pathways associated with the UPR and the initiation of protein translation, which is the rate-limiting step in translation, could regulate the acquisition of chemoresistance in TNBC cells [9]. Recent findings have demonstrated that inhibition of proteins involved in translation initiation, specifically the three members of the eIF4F cap-binding complex eIF4E, eIF4G, and eIF4A, promotes sensitization of tumor cells [10].

eIF4F binds 7-methylguanylate at the 5′ end of mRNA, thereby modulating protein translation. eIF4A carries out scanning and unwinds the secondary structures of several mRNAs at the 5′ untranslated region (5′-UTR), thus promoting binding to the small ribosomal subunit [11]. The assembly of the mature eIF4F complex is modified by the binding of programmed cell death protein 4 (PDCD4) to eIF4A via the MA-3 domain, which could suppress the selective translation of mRNAs [12]. PDCD4 activity is associated with the inhibition of cancer cell proliferation [13], and it is negatively regulated by the cascade of the mTORC1/p70S6K pathway [14]. Although PDCD4 is related to estrogen receptor status [15], its role in TNBC and its association with UPR remain unclear. PDCD4 and eIF4A may become tightly associated during tumor development, and also potentially during drug resistance. In fact, a previous study reported that the ubiquitination and degradation of PDCD4 could increase the migration and invasion of ovarian and endometrial cancer cells [16]. Moreover, enhancing eIF4A activity could promote the translation of oncogenes required for tumor cell growth and survival via the dysregulation of HER2 or FGFR1/2, leading to PI3K/Akt activation and RAS/ERK signaling [17]. 

In response to ER stress, PERK activation through the pro-survival Nrf2 pathway triggers an intricate transcriptional and translational regulatory response, which ultimately affects the cell cycle [18]. The transcription factor Nrf2 has been identified as a substrate of PERK kinase, and their interaction mediates the translocation of Nrf2 to the nucleus [18]. Moreover, a connection between the PERK/Nrf2 axis with PDCD4 has been proposed. PDCD4 overexpression in lung tumor cells has been associated with the suppression of the transcriptional activation of Nrf2 through its negative regulator, Keap1 [19]. ER stress adaptations in cancer cells are likely linked to a drug-resistant phenotype. This presumably occurs through the downregulation of tumor suppressor genes such as PDCD4 with an impact on eIF4A and the PERK/Nrf2 axis, thus promoting the survival of cancer cells. Additionally, the activation of IRE1 through X-box-binding protein 1 (*XBP1*) could also contribute to the acquisition of chemoresistance [20].

Doxorubicin (Dox) is a broad-spectrum chemotherapeutic anthracycline drug that was first isolated from *Streptomyces peucetius* spp. [21,22,23]. Dox is currently used alone or in combination with other drugs to treat blood cancers and solid tumors (e.g., breast and thyroid) [21,24]. Dox inhibits topoisomerase II, interrupting DNA transcription and replication. Moreover, this drug increases the generation of reactive oxygen species, which leads to oxidative damage in DNA, mitochondria, and cell membranes [21,22,23,25]. TAC [docetaxel (Taxotere), doxorubicin hydrochloride (Adriamycin), and cyclophosphamide] is used as a neoadjuvant therapy in TNBC treatment [26]. Patients who respond to neoadjuvant TAC therapy have shown an excellent prognosis [26]. However, a lack of Dox response may also occur. The acquisition of a chemoresistant phenotype requires tumor cells to undergo molecular, biochemical, and structural adaptations. During this transition, the activation of antioxidant systems, coupled with the expression of specific transporters and detoxifying enzymes associated with the deregulation of the UPR [27], could affect the migration and invasion capability of cancer cells. These hallmarks are linked to the over-activation of the focal adhesion kinase (FAK) pathway and matrix metalloproteinase-9 (MMP-9). Moreover, eIF4A-dependent FAK activity could also promote metastasis and chemoresistance. 

Epithelial-mesenchymal transition (EMT) is a phenomenon associated with an increase in the migratory and invasive capability of cancer cells, which is accompanied by a greater secretion of MMPs; a hallmark of metastasis. Molecules isolated from natural sources or dietary phytochemicals could inhibit these phenomena. For example, arctigenin, baicalin, and carnosol inhibit the signal transduction pathways mediated by TGF-β, the primary regulator of EMT, thus reducing tumor migration and invasion [28,29], with potential implications for FAK activity. Moreover, silvestrol-induced eIF4A inhibition reduces FAK phosphorylation, which is an important driver of cell proliferation [30]. Therefore, eIF4A might regulate FAK activity in TNBC.

Recent evidence has suggested the potential impact of terpene molecules on cancer development [31]. In a previous work, cryptotanshinone (Cry), a terpene molecule isolated from the root of *Salvia brandegeei*, was found to potentially regulate eIF4A [32]. Thus, natural products could inhibit the processes associated with tumor progression and the regulation of chemoresistance. Inhibitors that target specific translation factors in cancer and several diseases have shown promising potential. For example, inhibitors of eIF4E such as 4EGI-1, 4E1RCat, and 4E2RCat have shown anti-tumor effects in preclinical trials [33]. Likewise, Salubrinal (Sal) is a selective inhibitor of p-eIF2α phosphatase, modulating the PERK arm of UPR through p-eIF2α inactivation, which inhibits the binding of Met-tRNAi^Met^ to the 40S ribosomal subunit [32].

The development of chemoresistance requires the activation of specific molecular responses such as adaptive proteostasis. Low-density lipoprotein (LDL) dyslipidemia could accelerate the acquisition of chemoresistance in breast cancer, as reported in our previous studies [34,35]. Here, in vitro experiments were conducted using a cellular model of TNBC to understand the mechanisms that link the UPR and Dox-induced chemoresistance. Our results suggested that downregulation of PDCD4 could be one of the hallmarks of acquired chemoresistance, with PDCD4 playing a key role as a modulator of cellular responses associated with the PERK/Nrf2 pathway. Our findings also suggested that the PDCD4/eIF4A axis plays a pivotal role in the regulation of chemoresistance development, and, therefore, novel treatments could be developed by targeting this axis using plant-derived terpenes based in the structure of Cry. More importantly, concomitant treatment with Cry and Dox could inhibit the chemoresistant phenotype in MDA-MB-231 cells. Therefore, strategic disruption of protein translation initiation is an attractive therapeutic target for the treatment of TNBC.

## 2. Materials and Methods

### 2.1. Materials

Cell culture reagents were purchased from Thermo-Fisher (Carlsbad, CA, USA), tissue culture plates and other plastic materials were obtained from Corning Inc. (Corning, NY, USA). Salts and buffers were obtained from Merck (Darmstadt, Germany). Doxorubicin (Dox), MTT, salubrinal (Sal) and tunicamycin (Tum) were obtained from Merck. Antibodies anti-XBP1s and anti-BiP/GRP78 were purchased from Abcam (Cambridge, UK). Anti-β actin, anti-CD47, anti-PCNA, anti-eIF4A, anti-PDCD4, anti-c-Jun, anti-MMP-9, anti-Nfr2, anti-BECN1, anti-Mdm2, anti-PERK, anti-CHOP, anti-FAK, anti-p-FAK were obtained from Sta. Cruz Biotechnology (Dallas, TX, USA). 

### 2.2. Cell Culture

The TNBC cell line model MDA-MB-231 was acquired from American Type Culture Collection (ATCC, Manassas, VA, USA), accession number: HTB-26. Cell cultures were grown in DMEM medium supplemented with 10% fetal bovine serum (FBS), 10 U/mL penicillin, 10 µg/mL streptomycin, and 25 µg/mL amphotericin B. Cultures were maintained at 37 °C in a humidified atmosphere of 95% air and 5% CO_2_. The culture medium was changed every 3 to 4 days according to ATCC recommendations.

### 2.3. Chemoresistance Protocols

To generate a drug-resistant phenotype, MDA-MB-231 cells were seeded at a density of 0.8 × 10^6^ cells/dish in p100 cell culture dishes. In one protocol, throughout the cell treatment with Dox increasing concentrations (0–100 nM), a resistant variant was obtained during three months of treatment. These cells were cultured in a Dox-maintaining dose (15 nM) based on the protocol reported by Carlisi et al. 2017 [36]. In a second method, we evaluated a scheme of lethal Dox dose (0.5 µM) for 24 h, later, the Dox treatment was diminished to 12.5 nM for 60 days. The third protocol performed a Dox-increasing dose for 60 days (15–60 nM range). Cellular variants were evaluated by MTT assay. Experimental conditions were assessed in resistant variants compared with the parental cells. In another report, we considered the IC_25_ of the parental cells, and cell cultures were incubated with an initial dose of Dox 0.3 μM for 4 days and a recovery period of 20–30 days, this cycle was performed six times. This strategy has allowed defining the role of factor eIF4E on the expression of ABCC transporters during chemoresistance (personal communication).

### 2.4. Cell Viability

Cell viability was evaluated employing the MTT assay according to previous protocol [37]. Formazan crystals were dissolved in a lysis buffer containing SDS 20% and N,N-dimethylformamide 50% (pH 3.7) for 12 h at 37 °C. Optical density was measured at 570 nm using a microplate reader.

### 2.5. Western Blot (WB) Analysis

Cell cultures were maintained in proliferation until 90% of confluence. Next, cells were incubated under several treatments. Cells were washed with PBS, and lysed for 35 min at 4 °C with protein buffer lysis supplemented with protease and phosphatase inhibitors. Further to centrifugation at 4100× *g* for 10 min, the supernatant was recovered and the protein quantification was carried out using the bicinchoninic acid assay (BCA). Samples (25 μg/lane) from the protein fraction were analyzed by SDS-polyacrylamide gel electrophoresis (SDS-PAGE) on 10% gels and further transferred to PVDF membranes. Next, membranes were blocked with 5% nonfat milk in Tris-buffered saline 0.1% Tween-20 (TBS-T) for 1 h at 37 °C and incubated at 4 °C overnight with the corresponding primary antibody. Following washing with TBS-T, membranes were further incubated for 90 min at 37 °C with the corresponding horseradish peroxidase-conjugated (HRP) secondary antibodies. Later, membranes were washed with TBS-T, and the HRP activity was detected with the Immobilon Western kit (Millipore, MA, USA). Analysis of immunoblot films was made with the ImageJ software (NIH, Maryland, DC, USA).

### 2.6. Characterization of Proteins in Extracellular Media

Proteins BiP and XBP1s were characterized in extracellular media by WB. Further to Dox (500 nM) and Sal (25 µM) treatments, extracellular media were recovered and concentrated 10× times by centrifugation (4000 rpm) employing Amicon Ultra-15, 10 kDa disposals. Samples were processed by SDS-PAGE and WB. Loading controls were performed with Coomassie stain on PVDF membranes and β-actin in cellular lysates. 

### 2.7. Immunoprecipitation Assays

Characterization of the complex PDCD4/eIF4A was evaluated by co-immunoprecipitation assay under several schemes. For these immuno-precipitation assays, cellular fractions (300 µg) were incubated with the anti-eIF4A (1:400) for 2 h at 4 °C. Next, immune complexes were precipitated with Protein G Agarose Fast Flow (Millipore) for 12 h at 4 °C, according to a previous protocol [38]. Immuno-precipitated proteins were washed 3 times and suspended in Laemmli buffer, separated by SDS-PAGE gels, and transferred to PVDF membranes for WB analysis. This same process was corroborated, but now PDCD4 was immuno-precipitated, and eIF4A detection was made.

### 2.8. Nucleus Isolation

Nuclei separation was carried out using a sucrose (250 mM) and imidazole (3 mM) pH 7.4 buffer, supplemented with protease and phosphatase inhibitors. After the treatments, cells were scraped from culture dishes, and 21 passages were performed through a 22G syringe. For the recovery of nuclei, lysates were centrifuged at 3400 rpm for 15 min. Two fractions (supernatant and pellet) were lysed for 25 min at 4 °C, and both fractions (20 μg/lane) were analyzed by SDS-PAGE and transferred to PVDF membranes. Membranes were incubated with primary antibody, and after successive washes, membranes were incubated with specific secondary antibody. HRP activity was detected. β-actin was used as a loading control in several experiments. Lamin B1 and GAPDH were evaluated in the nucleus and cytoplasmic fraction, respectively.

### 2.9. Evaluation of Cellular Dox-Internalization

Considering the Dox fluorescent properties, this feature was used to quantify Dox-concentrations in the cells and supernatant medium under different experiments, both in resistant variants and parental cells.

#### 2.9.1. Cell Cytometer Assays

After 1 h of starvation, cell cultures were incubated under the different treatments in OptiMEM medium in 10 mm wells. Then, cell cultures were washed 2 times with PBS and recovered in a volume of 200 µL. Finally, in cellular characterization was used a Beckman-Coulter cytometer Cytoflex. Using the PC5.5 channel to record the Dox-associated fluorescence, 30,000 events were registered.

#### 2.9.2. Dox Quantification in Supernatant Media

After experimentation, supernatant media were recovered and centrifuged at 5000 rpm for 10 min to eliminate cellular debris. Later, the extracellular media were processed in a Cary Eclipse Fluorescence spectrophotometer (Agilent Inc., Santa Clara, CA, USA), considering Dox-fluorescent properties (λ*exc*: 470 nm; and λ*em*: 595 nm) [39]. 

### 2.10. Scratch-Wound Assay

After 2 h of starvation, cultures were washed twice with PBS. Next, cultures were treated with 12 μM mitomycin-C for 2 h to inhibit cell proliferation. Thus, culture dishes were scratch-wounded using a sterile 200-μL pipette tip and washed twice with PBS. Later, cells were exposed to the treatments for 36 h. Finally, cells were fixed and crystal-violet stained; the progress of cell migration into the wound was photographed using an inverted microscope coupled to a digital camera. Cell migration was evaluated by ImageJ software.

### 2.11. Cellular Invasion Assays

Experiments were performed with the modified Boyden chamber method in 24-well plates containing 12 cell culture inserts (8 μm pore size) (Corning, Inc., New York, NY, USA). Briefly, 30 μL of matrigel (Corning) was added into culture inserts and kept at 37 °C to form a semisolid matrix. After 2 h of starvation, cells were pre-treated for 2 h with 12 μM mitomycin-C, then cells were seeded into the upper chamber of the Boyden chamber at 1 × 10^5^ cells/well. Treatments were added to the lower chamber. Both the upper and lower chambers contained FBS-free DMEM. After 48 h of incubation, cells on the lower membrane surface were fixed in methanol for 5 min. Membranes were treated with triton 0.01% and stained with hematoxylin. Later, cells were photographed using an inverted microscope.

### 2.12. Zymography

Cells were incubated under different treatments, and the conditioned medium was collected. Volumes of 40 µL non-heated conditioned medium samples were mixed with 5× sample buffer (0.313 M Tris pH 6.8, 10% SDS, 50% glycerol, and 0.05% bromophenol blue) and loaded on 8% polyacrylamide gels copolymerized with gelatin (1% *w*/*v*). Gels were rinsed twice with 2.5% Triton X-100 and then incubated in a development buffer (50 mM Tris–HCl pH 7.4, 10 mM CaCl_2_, and 0.02% NaN_3_) for 40 h at 37 °C. Gels were fixed and stained with 0.25% Coomassie Brilliant Blue G-250 in 10% acetic acid and 30% methanol. Proteolytic activity was detected as clear bands against the background stain of the unprocessed substrate. Likewise, conditioned medium samples were processed by SDS-PAGE, further transferred to PVDF membranes, and MMP-9 WB was done.

### 2.13. Purification of Tanshinone Molecules

The root of *Salvia brandegeei* as part of the chaparral vegetation was collected in the coordinates 31°6′60″ N and 116°16′9″ W, Baja California, Mexico. The dry plant material was subjected to an extraction process by alcoholic maceration. Then, the raw extract was partitioned using solvents of increasing polarity, obtaining four partitions: n-hexane, methylene chloride, water, and butanol. The intermediate polarity extract (methylene chloride) was subjected to flash chromatography, using a mixture of n-hexane/ethyl acetate as eluent. Preparative chromatography on silica gel allowed the isolation of two secondary metabolites belonging to the terpene-tanshinone family: Cry (296.4 g/mol) and tanshinone IIA. Both molecules were identified and characterized by mass spectrometry and nuclear magnetic resonance spectroscopy, a previously reported method [32]. The Cry molecule was more abundant and evaluated in this work.

### 2.14. Isolation of LDL and Fluorescent Labeling

A human plasma sample was obtained from a healthy donor that signed informed consent. The protocol was designed and carried out according to the Declaration of Helsinki. In the first step, plasma density was adjusted to 1.019 g/mL with KBr and centrifuged at 345,500× *g* for 160 min using a S140-AT 2555 rotor, the VLDL and IDL-containing layer was discarded. Subsequently, density was adjusted to 1.053 g/mL and centrifuged at 377,000× *g* for 200 min; the upper fraction containing LDL was recovered. HDL was isolated, adjusting to a 1.21 g/mL density, and centrifuged at 377,000× *g* for 180 min. LDL preparation was dialyzed against 150 mM NaCl and filtered through 0.45 μm. LDL concentration was measured with the BCA method. The quality of the lipoproteins fraction was evaluated by apoB-100 and apoA1 characterization (data not shown).

LDL labeling was carried out with the dilC18 probe (D3911), which is incorporated in the phospholipid monolayer by incubation of 10 µL of the probe (2 mg/mL) for each 1 mg of protein-LDL for 18 h at 37 °C, obtaining dil-LDL. Solution density was adjusted to 1.053 g/mL and centrifuged at 377,000× *g* for 3 h to recover fluorescent lipoproteins as previously described [40,41]. The fraction was recovered and dialyzed against PBS1X.

### 2.15. Molecular Docking

eIF4A1 three-dimensional structure was obtained from the Protein Data Bank ID 5ZC9, which corresponds to the structure of human eIF4A1-ATP with a resolution of 2Å [42]. The structures of ligand molecules were obtained from the PubChem database [43], Cry (CID 160254) and rocaglamide (CID 331783). The protein structure was prepared by removing water and small molecules, leaving only the protein structure. Ligand and receptor were 3D-protonated, and energy minimization was performed using Molecular Operating Environment (MOE) software with default parameters under the AMBER89 force field. For the ligand, it generates different conformations by the use of a stochastic search in MOE default parameters.

The active site was predicted by employing the site finder option of the MOE software [44]. Molecular docking was set at default parameters for MOE software, and the pre-conformations were employed. For the interpretation of docking results, MOE identifies salt bridges, hydrogen bonds, hydrophobic interactions, sulfur-LP, cation-π, and solvent exposure and gives the S score. Ligand interactions with target proteins were predicted on the basis of the S score [45].

### 2.16. Small Interfering RNA (siRNA)

Cells were seeded at a density of 2 × 10^5^ cells/plates and incubated overnight in a standard growth medium without antibiotics. eIF4AI siRNA (h) (sc-40554) and control siRNA-A (sc-37007) were purchased from Santa Cruz Biotechnology (Santa Cruz, CA, USA). Transfection was performed according to the manufacturer’s protocol. After verifying the transfection efficiency by WB, experimentation with Dox and Cry was performed.

### 2.17. Overexpression of eIF4AI in MDA-MB-231 Variants

The human eIF4AI cDNA (gene accession number NM 001416.4) under the control of CMV-promotor in a pcDNA3.1(+) vector was acquired under the project ID: U533ZGE280-1, clone ID: HP4425A (GenScript, Piscataway, NJ, USA). Plasmid DNA was transformed into *E. coli* strain DH5α. Scrapings from colonies were used to inoculate 5 mL of culture medium LB containing 100 μg/mL ampicillin; these mixtures were grown overnight at 37 °C with agitation. Plasmids were purified from the resulting cultures using E.Z.N.A. Plasmid DNA Mini Kit I (Omega Bio-TEK) according to the manufacturer’s protocol and verified through digestion with restriction enzymes HindIII (GibcoBRL, MD) and BamHI (BRL, MD). The purity and yield of DNA were determined by Nanodrop analysis. Transfections were performed using Lipofectamine 3000 (Invitrogen) following the manufacturer’s instructions. Controls were evaluated by WB employing eIF4A monoclonal antibody (ID 2490). The effect of treatments with Dox (500 nM) and Cry (25 µM) was performed. 

### 2.18. eIF4AI Overexpression and Purification

The gene encoding for *eIF4AI* cDNA (NM 001416.4) under the control of CMV-promotor in a pcDNA3.1(+) vector was acquired under the project ID: U533ZGE280-1, clone ID: HP4425A (GenScript, Waltham, MA, USA). This vector encodes for a 10-His-tag protein and a PreScission Protease (GE, USA) cleavage site. The plasmid was transformed into *E. coli* cells strain Rosseta-Star (Novagen), harboring the pET19b-*eIF4AI* plasmid. Cultures were grown at 37 °C in 2XYT medium complemented with ampicillin (100 µg/mL) to reach an optical density (OD_600_) of 0.6, followed by IPTG (1 mM) induction and further incubation for 12–20 h at 37 °C. After harvesting by centrifugation, cells were disrupted by sonication. The supernatant was obtained and washed with 50 mM NaH_2_PO_4_, 300 mM NaCl and 10 mM imidazole buffer. Purification was performed by affinity chromatography (Ni-NTA Agarose resin QIAGEN, Germany). Column was washed with 20 column volumes of 20 mM imidazole, 50 mM HEPES-KOH (pH 7.6), and 600 mM NaCl buffer. The bound protein was eluted with 200 mM imidazole, 20 mM HEPES-KOH, and 100 mM NaCl buffer. The His-tag was removed from *eIF4AI* with PPS (PreScission Protease), the purified protein was dialyzed against PBS 1× buffer pH 8.0 following the manufacturer’s protocol. PPS was eliminated by glutathione Sepharose (Merck, Darmstadt, Germany). eIF4AI homogeneity was confirmed by SDS-PAGE with coomassie blue staining and WB. All reactants were molecular biology grade and ultrapure water.

Fluorescence measurements were performed with a Cary Eclipse fluorometer, using the following parameters, a scan from 250 to 350 nm at 25 °C in a synchronous mode. We evaluated the interaction protein-ligand, under eIF4A1 (12 µM) and cryptotanshinone (0–80 μM). Solutions were homogenized and incubated for 5 min at 25 °C, measurements were performed in a quartz cell with a path length of 1.0 cm and 500 mL volume. 

### 2.19. Statistical Analysis

Data are expressed as mean ± SD. The statistical analyses were conducted with one-way ANOVA. In MTT assays data are expressed as mean ± SD.

## 3. Results

### 3.1. Association between UPR and Dox-Induced Chemoresistance

**Figure 1:**
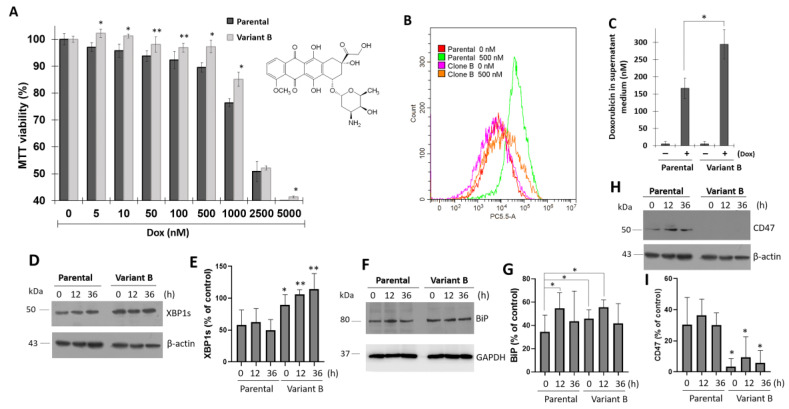
Chemoresistance induced by Doxorubicin (Dox) is associated with UPR activation. (**A**) MTT assay in parental and variant B cells, mean values are presented (n = 6, mean ± SD), * *p* < 0.001, ** *p* < 0.05, respect to variant cells. Dox structure is shown. (**B**) Cytometer analysis under 500 nM of Dox-treatment in parental cells and variant B. Under the same condition, fluorometer analysis of Dox in supernatant medium (**C**), mean values are presented (n = 3, mean ± SD), * *p* < 0.05 compared to the control. (**D**) WB analysis of XBP1s in a Dox 500 Nm temporal curse (0–36 h), densitometry analysis of XBP1s is shown (**E**). Under the same temporal curse, evaluation of BiP (**F**,**G**) and CD47 expression (**H**,**I**). In densitometry analysis of proteins, mean values are presented (n = 3, mean ± SD) and expressed as % of control, * *p* < 0.05, ** *p* < 0.01. β-actin and GAPDH were used as a loading control.

TNBC is a highly aggressive and chemoresistant type of cancer [46], and MDA-MB-231 cell cultures represent an optimal model to evaluate the mechanisms that drive chemoresistance. Therefore, we characterized several protocols to obtain Dox-induced chemoresistant MDA-MB-231 cells (TNBC cells). Specifically, the cells were treated with a lethal Dox dose (0.5 µM) for 24 h, followed by a gradual decrease in Dox dose for 60 days until a dose of 12.5 nM was reached, which led to the generation of a variant that will hereinafter be referred to as “variant B.” As demonstrated by the MTT assay, variant B showed a resistant phenotype (Figure 1A). Upon evaluating Dox internalization through flow cytometry analysis, variant B exhibited a decrease in Dox internalization (Figure 1B). Moreover, higher concentrations of Dox were detected in the supernatant medium of variant B cells compared with the parental cells according to spectrofluorometric characterization (Figure 1C).

To gain insights into the role of the UPR in the chemoresistance process, cells were treated with Dox (500 nM) in a time-dependent way (0–36 h). Our findings suggested that transcription factor XBP1s is differentially regulated in variant B, as demonstrated by a significant increase in XBP1s protein expression (Figure 1D,E). When XBP1s becomes activated, this transcription factor translocates to the nucleus and triggers the expression of chaperones, lipid synthesis, and ER-associated degradation genes [47]. In variant B, we identified a strong activation of binding immunoglobulin protein (BiP), a UPR chaperone master regulator (Figure 1F,G). This was consistent with one of our previous studies, in which metabolic inflammation induced ER stress [48]. Chaperones such as heat shock proteins are associated with key cancer hallmarks such as cell proliferation, invasion, and metastasis [49]. Furthermore, this phenomenon was associated with a reduced expression of CD47 (Figure 1H,I), a target gene related to migratory and invasion hallmarks [50]. Although increased invasion is a common feature of drug resistance, recent evidence has demonstrated the occurrence of the opposite effect. Specifically, a previous study reported a reduction in the migratory capacity of docetaxel-resistant prostate cells compared to the parental cells [51]. Whether this behavior can be promoted or inhibited may depend mainly on the resistance mechanisms of a specific cell type and the tumor microenvironment. Therefore, migratory capacity could have been impaired in variant B.

### 3.2. Cell Migratory Capacity Is Associated with the Disruption of the PDCD4 Tumor Suppressor Gene

**Figure 2:**
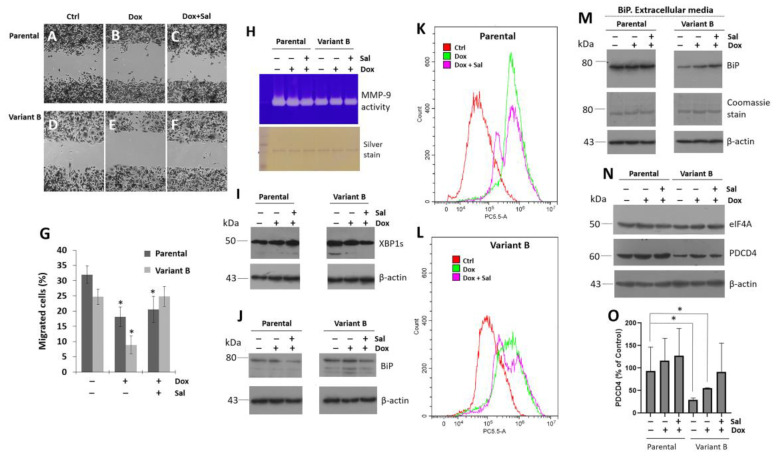
Chemoresistant cells showed a reduced migratory capacity coupled with PDCD4 dysregulation. Representative images of the wound-healing assay in parental cells corresponding to control (**A**), Dox treatment (500 nM) (**B**), and concomitant Dox (500 nM) and Sal (25 µM) treatment (**C**). The same assay in variant B, control (**D**), Dox treatment (**E**), and concomitant Dox and Sal (**F**). (**G**) Wound-healing results expressed as percentage respect to controls in parental and variant B cells. Mean values are presented (n = 3, mean ± SD); * *p* < 0.01 respect to controls. (**H**) Under the same conditions, MMP-9 secretion was analyzed on a conditioned medium using gelatin-substrate gels. The silver stain was employed as a loading control. WB analysis of XBP1s (**I**) and BiP (**J**) on parental cells and variant B, under treatment with Dox (500 nM) and Sal (25 µM). Dox characterization by cytometer analysis in parental cells (**K**) and variant B (**L**) under Dox (500 nM) and Sal treatment (25 µM). (**M**) BiP expression in extracellular media of parental cells and variant B under Dox (500 nM) and Sal treatment (25 µM). β-actin and Coomassie stain on PVDF membranes were used as controls. (**N**) At the same conditions, WB of eIF4A and PDCD4. (**O**) Densitometry analysis of PDCD4, results are reported as mean ± SD (n = 3) and expressed as % of control; * *p* < 0.05 respect to control. β-actin was used as a loading control in WB experiments.

Scratch-wound assays were conducted to characterize cell migration, as this is one of the main hallmarks of cancer onset and progression. The resistant variant B exhibited a marked decrease in migratory capacity compared to the parental cells. This was consistent with the UPR deregulation observed in resistant variant B (Figure 2A,D), according to CD47 expression. However, Dox treatment (500 nM) decreased the migratory capability of both variants (Figure 2B,E). Sal (25 µM) ameliorated the chemoresistance-inducing effects of Dox (Figure 2C,F), as suggested by the results of a semi-quantitative analysis (Figure 2G). Additionally, MMP-9 activity was also evaluated in extracellular media, and our findings demonstrated a decrease in MMP-9-mediated collagen degradation in variant B. Gel silver stain was used as a loading control in our experiments (Figure 2H).

Next, the disruption of the IRE1 arm by XBP1s in the chemoresistant variant B was confirmed. Our results suggested a connection between the activation of XBP1s, the expression of chaperone BiP, and the effect of Sal-treatment in variant B (Figure 2I,J). The opposite was observed in another variant with a sensitive phenotype (variant F). Specifically, the cells did not exhibit XBP1s overexpression and their Dox internalization levels were similar to those of the parental cells (Supplementary Figure S1). These results indicated the induction of an adaptive UPR in chemoresistant variant B. As expected, when Dox intracellular levels were evaluated by flow cytometry, a high Dox signal was observed in the parental cells compared to variant B (Figure 2K,L), which could explain the chemoresistance of the latter. Sal treatment induced a slight reduction in intracellular Dox signals in both models (Figure 2K,L), which was associated with the protective results described above.

Moreover, our findings broadened the potential association between BiP and the ER stress response in instances of cell damage. Specifically, increased secretion of BiP has been associated with the depletion of ER calcium [52]. Here, we detected the presence of BiP in the extracellular medium of the parental and variant B cell cultures (Figure 2M). This response was cell-specific, with BiP levels being lower in the resistant variant. Tunicamycin (Tum), a potent ER stress inducer, was used as a control (Supplementary Figure S2). We have previously reported the importance of chaperones such as PDI in the process of chemoresistance [48], as well as the potential involvement of the transcription factor XBP1s, a transducer of the IRE1 arm (Supplementary Figure S3). Therefore, we are currently elucidating the role of these proteostasis elements in the extracellular environment in conjunction with BiP (personal communication). Given that protein translation is a crucial mechanism associated with the activation of the UPR [53] and potentially also chemoresistance, we evaluated the expression of eIF4A, a key translation initiation factor. No significant changes in eIF4A expression were observed in the parental and variant B cells even under Dox and Sal exposure (Figure 2N). The basal expression of the tumor suppressor gene PDCD4 remained consistently high in parental cells and our data suggested that cell division control mechanisms were still likely present. Although mainly Dox treatment could promote mechanisms that sensitize the chemoresistance, PDCD4 expression was not modified in the parental cells. In contrast, PDCD4 was markedly downregulated in variant B (Figure 2N,O).

### 3.3. Characterization of the Role of the Tumor Suppressor Gene PDCD4

**Figure 3:**
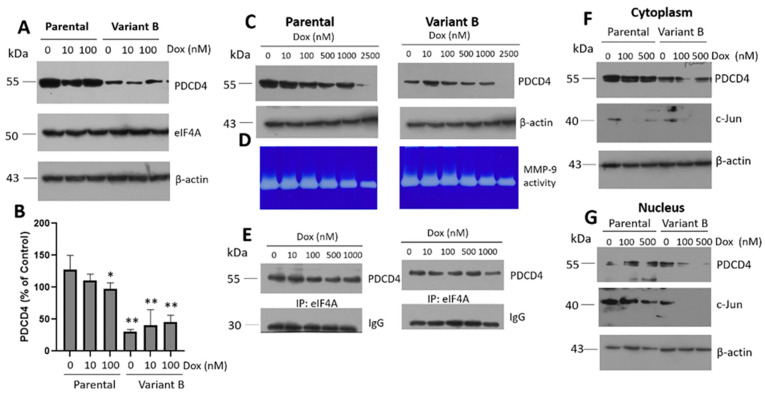
Doxorubicin (Dox) induces the disruption of PDCD4/eIF4A complex. (**A**) WB of PDCD4 and eIF4A under 10 and 100 nM Dox treatment in parental cells and variant B. (**B**) Densitometry analysis of PDCD4, results are reported as mean ± SD (n = 3) and expressed as % of control; * *p* < 0.1, ** *p* < 0.001 respect to control of parental cells. (**C**) PDCD4 expression is affected by increasing doses of Dox (0–2500 nM) in both, parental and variant B cells. Under the same conditions, MMP-9 secretion was analyzed on a conditioned medium using gelatin-substrate gels (**D**) and the interaction of PDCD4/eIF4A complex (**E**). (**F**,**G**). Cellular localization of PDCD4 and c-Jun in parental and variant B cells, under increasing concentrations of Dox (0–500 nM). β-actin was used as a loading control, and Lamin B1 in nucleus fractions.

To the best of our knowledge, no previous studies have characterized the role of PDCD4 on ER stress and its connection with cancer chemoresistance. PDCD4 deficiency has only been associated with low ER stress levels in a murine model [40]. The reduction of PDCD4 in variant B could alleviate ER stress or promote adaptations related to its tumor suppressor function. Therefore, we next sought to characterize the expression of PDCD4 and eIF4A under Dox treatment (0–100 nM). Specifically, we characterized the basal expression of eIF4A in both variants and confirmed that PDCD4 was exclusively downregulated in variant B (Figure 3A,B). Furthermore, upon exposing the cells to a broad range of Dox concentrations (0–2500 nM), PDCD4 was critically downregulated in both variants at 2500 nM (Figure 3C), which was possibly associated with extensive cellular damage. Under the same conditions, Dox dose-dependently induced a gradual reduction in MMP-9 activity, which coincided with the impairment of cellular migration and invasion (Figure 3D). In turn, this confirmed the occurrence of cellular damage, with these effects being more evident in parental cells. Finally, we evaluated the interaction between PDCD4 and eIF4A by co-immunoprecipitation. In both variants, Dox decreased the protein interaction signal in a dose-dependent manner (Figure 3E).

Although low PDCD4 expression levels were observed in variant B, these effects were not related to changes in eIF4A interaction. PDCD4 expression was impaired in variant B, and previous studies have reported that chemoresistance is linked to PDCD4 depletion [54]. Therefore, the regulation of eIF4A expression and the downregulation of PDCD4 are critical during chemoresistance, as these mechanisms enable the maintenance of cellular latency. In this regard, PDCD4 expression in breast cancer cells could thus be regulated by non-coding RNAs, ubiquitin-proteasome activity, and inflammation [55].

Our study also characterized the cellular localization of PDCD4 in the cytoplasm and nucleus fractions under different Dox concentrations (0–500 nM) (Figure 3F,G). PDCD4 appeared to be mainly localized in the cytoplasmic fraction of both the parental and variant B cells, suggesting that the activity of this protein was primarily cytosolic. In cancer cells, decreases in nuclear PDCD4 have been correlated with tumor progression [56]. In line with this notion, MMP-9 expression could depend on the transcription factor AP-1, a heterodimer composed of c-Fos and c-Jun. Our results indicated that Dox dose-dependently decreased the expression of c-Jun in the nucleus extracts of both cellular variants (Figure 3F,G), which possibly explains the changes in MMP-9 secretion in the extracellular medium. Although a regulatory mechanism mediated by the AP-1 complex and, therefore c-Jun is carried out, other mechanisms could have also been involved, including the activity of the tumor suppressor DACH1 (Dachshund Homolog 1) [57], miR-194-5p [58], and decreases in sirtuin 6 [59]. Moreover, a potential association between PDCD4 and c-Jun signaling was suggested by the expression of miR-21, which promotes the migration, invasion, and angiogenic capacity of renal carcinoma cells [60]. 

### 3.4. Small Molecule Treatment Can Desensitize the Chemoresistance of BC Cells

**Figure 4:**
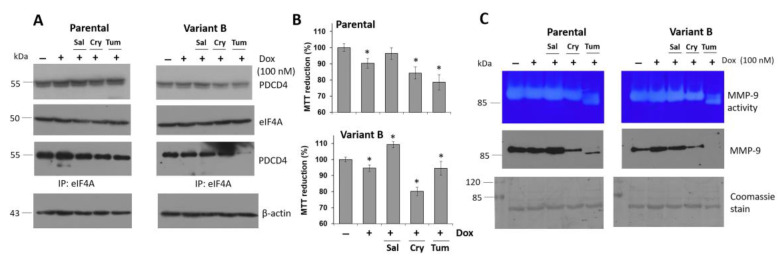
Chemoresistance is regulated by a terpene-derived molecule and ER-stress inducer compound. (**A**) Effect of concomitant Dox (100 nM) treatment with Salubrinal (Sal) (25 µM), Cryptotanshinone (Cry) (25 µM), and Tunicamycin (Tum) (1 µg/mL) on the expression of eIF4A, PDCD4, and characterization of protein interaction. (**B**) Employing the same conditions, cellular viability was evaluated in parental and variant B cells under the Dox treatment with Sal, Cry, and Tum. Mean values are presented (n = 6, mean ± SD), * *p* < 0.005 with respect to the control. (**C**) MMP-9 secretion was analyzed on supernatant medium using gelatin-substrate gels and the MMP-9 expression by WB. Membranes were processed by Coomassie stain as a loading control.

To counteract the chemoresistance of BC cells and elucidate the role of the eIF4F complex, the therapeutic and sensitizing effects of several molecules were evaluated under a low dose of Dox (100 nM) to avoid the side effects associated with high Dox doses [61], and proposing a potential sensitizing effect of candidate molecules. Considering the regulatory impacts of Cry on eIF4A [32], we next isolated this terpene from the root of *Salvia brandegeei*. Tum was used as a control. In our first experiment, we confirmed the decrease of PDCD4 expression in variant B (Figure 4A). Likewise, Cry treatment decreased the expression of the PDCD4-eIF4A complex in both variants. Moreover, Cry (25 µM) and Dox (100 nM) synergistically affected cell viability (Figure 4B), which was likely due to an interaction between Cry on eIF4A, which in turn affects PDCD4/eIF4A interactions. This effect was not observed under Sal treatment (25 µM) (Figure 4A,B), possibly associated with the inability to eIF4A binding. Finally, we examined the effect of Tum treatment (Figure 4A), and variant B was found to be more resistant to Tum than the parent cells (Figure 4B). 

Next, we evaluated the impacts of the aforementioned treatments coupled with Dox on MMP-9 expression, which is associated with cell invasion. Cry and Tum treatments + Dox evidently affected MMP-9 activity (Figure 4C), as demonstrated by the quantification of MMP-9-mediated collagen degradation (Supplementary Figure S4A). Likewise, the cytotoxicity of Cry and Tum was confirmed by optical microscopy in both variants (Supplementary Figure S4B–G), which was manifested as a reduction in cell density. Western blot analysis of MMP-9 in the extracellular media confirmed the zymogram results under Cry and Tum treatments coupled with Dox. Specifically, we identified changes in the glycosylation pattern of MMP-9 (90 kDa) in the samples treated with Tum, an inhibitor of protein N-glycosylation, with the parental variant showing a lower protein band of approximately 85 kDa, as well as the same molecular weight band on zymogram gels (Figure 4C). Similar patterns have been reported for other proteins such as cyclooxygenase-2 under inflammatory conditions induced by lipopolysaccharide treatment [41]. Sal incubation did not induce significant changes in MMP-9 activity and expression. In fact, MMP-9 levels were slightly higher compared to the controls, which confirms the protective properties of Sal.

### 3.5. Relevance of PERK/Nrf2 and Regulation by Terpene-Derived Molecules

**Figure 5:**
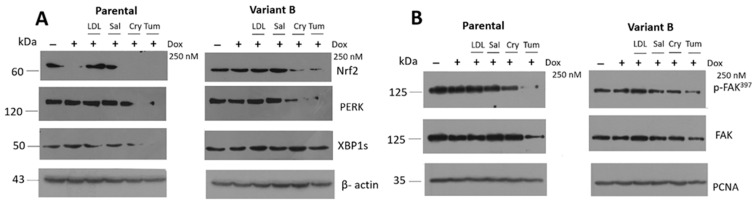
Doxorubicin (Dox) chemoresistance is associated with PERK/Nrf2 activation and regulated by cryptotanshinone (Cry). (**A**) Expression of Nrf2 under the Dox treatment (250 nM) and concomitant LDL (10 µg/mL), Sal (25 µM), Cry (25 µM), and Tum (1 µg/mL) in parental and variant B cells. In this experiment, XBPIs role was confirmed in variant B. β-actin was used as a loading control. (**B**) Under the same conditions, characterization of FAK pathway, evaluated by the expression of FAK and its phosphorylation at Tyr-397 (p-FAK^397^). PCNA was used as a loading control.

We evaluated the effect of small molecules on the PERK-signaling by the expression of pro-survival Nrf2, results suggest an increased activation of the PERK arm in variant B (Figure 5A), which was likely a compensatory cellular response. Nrf2 triggers the expression of genes involved in stress resistance and mitochondrial biogenesis [62]. Critically, Nrf2 activation was suppressed under concomitant Dox (250 nM) + Cry (25 µM) and Tum (1 µg/mL) treatments. In lung tumor cells that overexpressed PDCD4, the transcriptional activation of Nrf2 was inhibited [19]. Importantly, these findings were consistent with our observations in chemoresistant variant B, wherein PDCD4 downregulation coincided with Nrf2 activation. Moreover, an increase in the activation of XBP1s in variant B was also confirmed as an experimental control (Figure 5A).

Several phenomena such as migration, adhesion, and proliferation have been associated with the overexpression of UPR-associated genes such as BiP, PERK, and the target of cellular adhesion FAK in a colorectal cancer model [50]. Similarly, FAK activation (p-FAK^397^) was differentially regulated in our study (Figure 5B), with parental cells exhibiting higher levels of FAK expression and p-FAK activation, suggesting a higher migration activity (Figure 2) and possibly a higher invasion capacity. However, Cry treatment significantly diminished p-FAK in both variants (Figure 5B). Our previous results indicated that chemotherapy resistance could modulate adaptive mechanisms associated with the UPR in cancer cells. This phenomenon coincided with MMP-9 secretion (Figure 2H and Figure 4C) and FAK activation (Figure 5B), suggesting a crucial role of proteostasis and targets regulating translation initiation such as the translation initiation factor eIF4A.

### 3.6. Regulation of Cell Invasion by Cry Treatment

**Figure 6:**
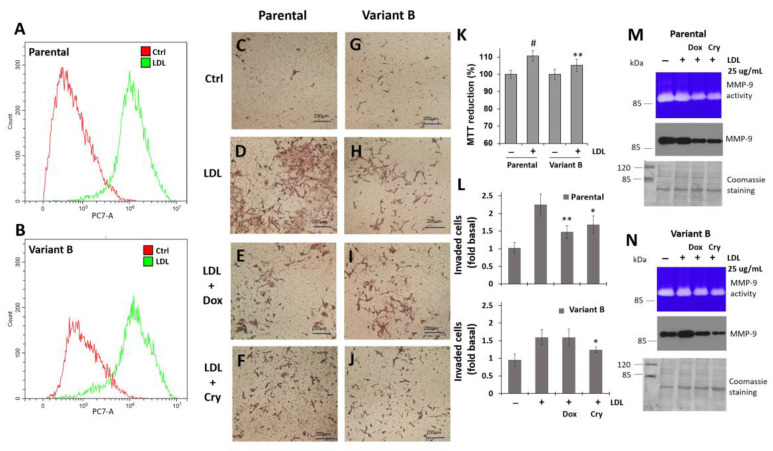
Cry treatment inhibits LDL-induced cellular invasion. Dil-LDL internalization by cytometer analysis in parental (**A**) and variant B (**B**) cells. Representative images of invasion experiments were obtained by optical microscopy, employing Boyden chambers. In parental cells, representative image corresponding to control (**C**), LDL treatment (20 µg/mL) (**D**), LDL and Dox (500 nM) (**E**), LDL and Cry (25 µM) (**F**). The same assays showed in variant B, control (**G**), LDL treatment (**H**), LDL and Dox (**I**), LDL and Cry (**J**). Bars correspond to 200 µm. (**K**) Cellular viability in the two variants under the LDL treatment (20 µg/mL). Mean values are presented (n = 3, mean ± SD), # *p* < 0.001, ** *p* < 0.01 with respect to control. (**L**) Semi-quantitative analysis of invasion experiments in parental and variant B cells, graphs are expressed as the fold of control. Mean values are presented (n = 3, mean ± SD), * *p* < 0.05 with respect to LDL treatment, ** *p* < 0.01 with respect to LDL treatment. Effect of Dox (500 nM) and Cry (25 µM) on LDL induced-MMP-9 secretion in parental (**M**) and variant B (**N**) cells, activity was analyzed on the supernatant medium using zymogram gels and the expression of MMP-9 by WB. Membranes were processed with Coomassie stain as an experimental loading control.

Considering the differential regulation of FAK in parental cells and variant B, we next characterized the invasion capacity of BC cells via the Boyden chamber method. We previously reported the critical impact of intracellular lipid pathways on BC development. Specifically, LDL cholesterol could promote cell invasion by triggering epithelial-mesenchymal transition [34,35]. Therefore, LDL treatments were performed to evaluate the putative role of LDL as a potentiator of cellular invasion. Our flow cytometry analyses demonstrated the occurrence of LDL endocytosis in parental and variant B cells (Figure 6A,B). In the control condition, the invasion capability of both clones remained at basal levels (Figure 6C,G).

LDL treatment increased the invasion capacity of the parental cells (Figure 6D) compared with the variant B cells (Figure 6H), which was attributed to an increase in the expression and activation of FAK according to our previous results. Critically, we observed an increase in cellular viability promoted by LDL treatment on both variants (Figure 6K). However, the concomitant Dox (Figure 6E,L) and Cry (Figure 6F,L) treatments significantly decreased the invasion capacity of the parental cells. Dox treatment did not affect the invasion capacity of variant B (Figure 6I,L), whereas Cry treatment did (Figure 6J,L). Moreover, to gain insights into the mechanisms through which Dox (500 nM) and Cry (25 µM) modulate LDL-induced invasion, the critical MMP-9 target was characterized. Our findings indicated that LDL promoted an increase in MMP-9 secretion and activity (Figure 6M,N) in both the parental and variant B cells. In contrast, Cry treatment markedly decreased MMP-9 secretion in both variants (Figure 6M,N). Collectively, our results suggested that LDL treatment promotes the cellular invasion capacity of both parental and variant B cells, whereas Cry exerts the opposite effect on this key cancer hallmark by inhibiting MMP-9.

### 3.7. Modulation of eIF4A on Oncogenic Factor FAK

**Figure 7:**
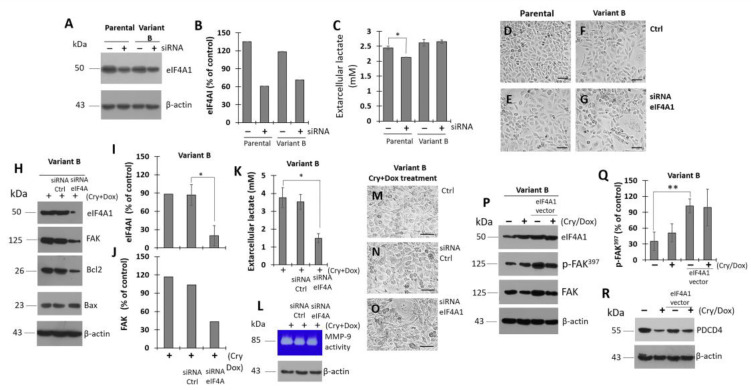
Regulation of eIF4A on FAK is inhibited by concomitant treatment of Dox and Cry. Efficiency of siRNA incubation (80 nM) on the expression of eIF4A1 in parental and variant B at 24 h. (**A**) WB of eIF4A1 in parental cells and variant B and densitometry analysis of eIF4A1 (**B**); (**C**) under the same conditions, lactate concentrations in supernatant media, results are reported as mean ± SD (n = 3), * *p* < 0.01, respect to control. Optical microscopy images corresponding to control (**D**) and siRNA-eIF4A treated parental cells (**E**); for variant B, control cells (**F**) and siRNA-eIF4A1 treatment (**G**). In controls, the transfection vehicle was evaluated. Bars correspond to 200 µm. (**H**) Effect of siRNA-eIF4A1 on the expression of FAK, Bcl2 and Bax under the concomitant treatment of Cry (25 µM) and Dox (500 nM) for 60 h in variant B; densitometry analysis of eIF4A1 (**I**) and FAK (**J**), results are reported as mean ± SD (n = 3) and expressed as % of control; * *p* < 0.01 respect to control. Under the same transfection conditions and concomitant Cry and Dox treatment, evaluation of lactate in supernatant media (* *p* < 0.01 respect to control) (**K**), MMP-9 activity (**L**), and representative cellular images of control (**M**), siRNA control (**N**), and eIF4A1-siRNA (**O**). Bars correspond to 200 µm. (**P**) Effect of the eIF4A1 overexpression on FAK expression and activity (p-FAK^397^) employing an eIF4A1 vector (GenScript, ID: U533ZGE280-1). Results showed the effect of Cry (25 µM) and Dox (500 nM) treatment for 24 h. (**Q**) densitometry analysis of p-FAK^397^, results are reported as mean ± SD (n = 3) and expressed as % of control; ** *p* < 0.05 respect to control. (**R**) Under the same conditions, WB of factor PDCD4. In panels A, H, L, P, and R, β-actin was used as a loading control.

Upon exploring the impact of eIF4A1 (the main isoform) on the chemoresistance process and considering the regulatory role of therapeutic small molecules such as Cry, we next performed experiments to evaluate the effects of modulating eIF4A1 expression. Using siRNA (80 nM), eIF4A1 was successfully knocked-down after treating parental and variant B cells for 24 h (Figure 7A,B). Specifically, eIF4A expression was decreased by 55% and 40% in the parental and variant B cells, respectively (Figure 7B). As a complementary assay, lactate was quantified as an indicator of energetic metabolism; primarily, the siRNA effect takes place in parental cells (Figure 7C). Several studies have reported that cancer cells exhibit an increased glucose uptake and lactate secretion due to metabolic abnormalities, which is referred to as the Warburg effect [63]. Here, siRNA-affectation in lactate secretion was coupled with modifications of cellular density mainly in parental cells (Figure 7D,E) compared to variant-B cells (Figure 7F,G).

To gain insights into the regulation of chemoresistance and the role of eIF4A, we next focused on the characterization of the chemoresistant variant B. Our data suggested that concomitant Cry (25 µM) and Dox (500 nM) treatment (Figure 7H) resulted in an almost complete knockdown of eIF4A, reaching a residual expression level (22%) compared with the control (Figure 7I). Critically, this result was consistent with the expression of FAK (Figure 7H,J). Moreover, eIF4A knockdown was linked to cell damage, as evidenced by changes in lactate levels (Figure 7K), MMP-9 activity (Figure 7L), and optical microscopy (Figure 7M–O). Additionally, eIF4A1 was overexpressed to corroborate the association between eIF4A and FAK. eIF4A overexpression was achieved after 25 h of treatment (Figure 7P). Importantly, eIF4A levels were correlated with FAK expression and activation (Figure 7P,Q). Afterward, we confirmed the effect of Cry (25 µM) and Dox (500 nM) treatment on eIF4A expression. Based on our previous results, we identified an inverse association between PDCD4 and eIF4A (Figure 7R). Therefore, our findings confirmed that eIF4A, a regulator of protein translation, is crucial for proteostasis.

### 3.8. Cryptotanshinone Mechanism Is Mediated by eIF4A Interaction

**Figure 8:**
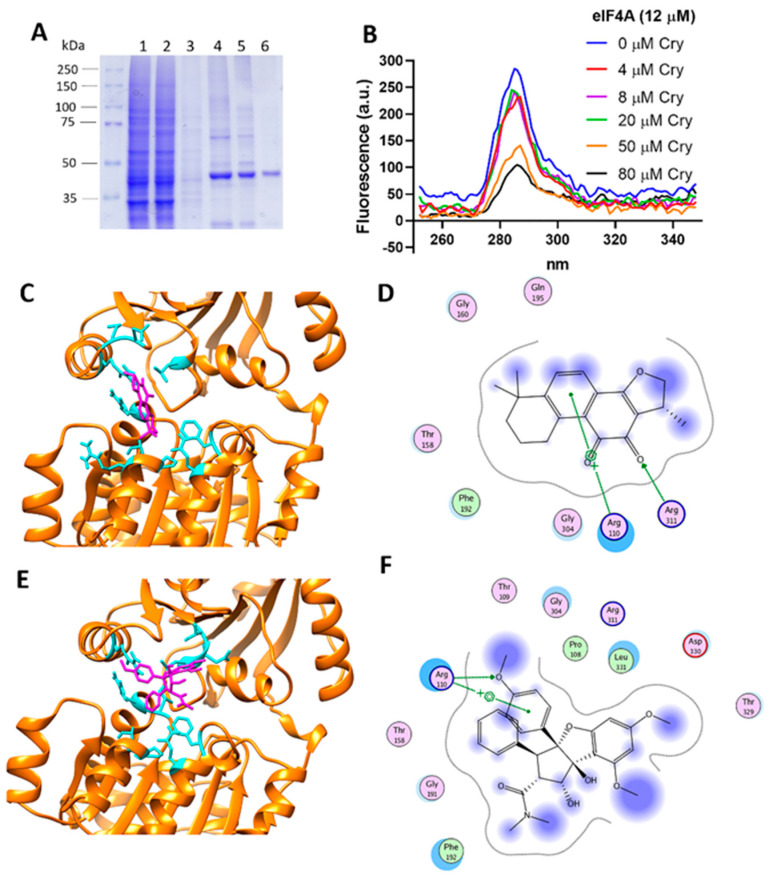
Cryptotanshinone (Cry) binds to translation initiation factor eIF4A1. (**A**) SDS-PAGE electrophoresis of eIF4A1 purification showing the affinity chromatography results. Lane 1: overexpression of eIF4A1; lane 2, flow-through fraction; lane 3 washing buffer with an imidazole 20 mM, lane 4 purified protein, Lane 5 and 6: His-tag cleavage with PPS after 16 h incubation. Protein of lane 6 was used in the experiments. (**B**) Binding experimentation evaluated by a synchronic mode fluorescence. A scan from 250 to 350 nm at 25 °C is showed, under eIF4A1 (12 µM) and Cry (0–80 μM) incubation. (**C**) Representative snapshot corresponding to the crystallographic structure of the eIF4A1⋅ATP PDB ID: 5ZC9 (yellow) under the Cry (magenta) interaction, obtained by molecular docking. Interaction showed a −8.19 S value. (**D**) Residues that define the eIF4A/Cry interactions. (**E**) As a control, a representative snapshot of the eIF4A1⋅ATP (yellow) under the RocA (magenta) interaction. Interaction showed a −10.04 S value. (**F**) Residues that define the eIF4A/RocA interactions.

We next sought to characterize the regulatory effect of Cry on eIF4A (Figure 8). Therefore, the overexpression of eIF4A was evaluated in several strains of *E. coli* under a variety of experimental conditions. The BL21 (DE3) pLysS, BL21 (DE3), and Rosetta-Start *E. coli* strains harboring plasmid pET-19b-mod-eIF4A were examined to select the optimal host for protein production. The Rosetta-Star strain had the best protein production yield. Proteins were obtained from the soluble fraction and purified by affinity chromatography, after which the PreScission protease was used to remove His tags (Figure 8A). Our process was validated through western blot analyses in several samples, confirming a pure fraction corresponding to eIF4A1. The purity of the isolated soluble protein was higher than 90% (Figure 8A). Therefore, we next performed protein-ligand interaction assays using a fluorescence-based method. Purified eIF4A protein (12 µM) and Cry (0–80 µM) were incubated at 25 °C for fluorescent synchronic characterization, and an emission peak was detected near 285 nm. Incubation with Cry resulted in a quenching phenomenon, suggesting that Cry interacted with eIF4A1 (Figure 8B). Particularly, a critical reduction in fluorescence values was observed at a Cry concentration of 20 µM and quenching was evident at a Cry/eIF4A molar ratio of 4:1.

Based on the evaluation of the crystal structure of the human eIF4A1-ATP-rocaglamide (RocA)-polypurine RNA complex (PDB: 5ZC9), our docking results indicated that the Cry interaction site was highly similar to the Rocaglamide (RocA), a selective eIF4A inhibitor [64]. Specifically, RocA and Cry shared several interaction residues including Arg_110_, Thr_158_, Phe_192_, and Gly_304_ (Figure 8C–F), thus explaining their interaction with eIF4A. Our analyses indicated that the Cry binding site is similar to the RocA interaction domain on the eIF4A structure, thus inhibiting the RNA binding capability of eIF4A [47]. The Phe_192_ and Thr_158_ residues could establish hydrophobic interactions, whereas Arg_110_ and Arg_311_ promoted ionic interactions on the Cry structure (Figure 8D). In turn, these interactions are critical to maintaining a high binding capability, and explain the changes in the spectroscopic properties of eIF4A1 (Figure 8B). According to our molecular docking results, the S score of Cry was lower (−8.19) than that of RocA (−10.04). Moreover, crystallographic data suggested the critical role of hydrogen bonds and strong π-π and CH/π interactions between RocA-Gln_195_ and RocA-G_8_, which significantly contributed to complex formation, as well as the stabilizing role of Phe_192_ [64]. Critically, both Gln_195_ and Phe_192_ could stabilize the Cry interactions in the eIF4A1 structure (Figure 8C,D). Therefore, our results suggest that Cry interacts with the RNA-binding domain of eIF4A1, which could serve as a basis for the development of novel therapeutic molecules based on the structure of Cry.

## 4. Discussion

The accelerated proliferation of tumor cells combined with an inflammatory microenvironment leads to an impairment of protein synthesis. In these cases, the activation of UPR arms has been found to drive chemoresistance in several types of tumors [65]. Our results confirmed an association between UPR and chemoresistance. In the chemoresistant variant B, we observed changes in the transcription factors XBPIs and Nrf2 associated with IRE1 and PERK arms of UPR, respectively. Although a previous study characterized XBP1s in cells exhibiting the luminal/ER+ BC subtype [66], the role of XBP1s in TNBC models has not been described in detail. Moreover, chaperone BiP overexpression has been implicated in cancer cell survival, proliferation, migration, and chemoresistance [67]. Additionally, the silencing of chaperone BiP can decrease the efflux activity of ABC transporters and the antioxidant response, thereby impairing the chemoresistant phenotype [68]. This evidence supports the results described in chemoresistant variant B, and, therefore, the UPR and chaperones may be contributing factors to the acquisition of Dox-induced chemoresistance. 

Sal has been evaluated as a promising anti-neoplastic therapy. In previous reports, Sal treatment increased cell death in BC cells. This mechanism could be dependent on the maintenance of p-eIF2α phosphorylation, thereby blocking protein translation [69]. Likewise, Sal treatment enhanced the radiation sensitivity of estrogen receptor cells [70]. Notably, we observed the opposite response in Sal-treated chemoresistant TNBC cells, which was attributed to compensatory mechanisms that can lead to a subtle inhibition in protein translation. A previous study reported that the anticancer properties of Sal are mediated by an upregulation of PDI, a multifunctional redox chaperone of the ER [71]. Likewise, Sal reduced the apoptotic CHOP levels induced by lithocholic acid in prostate cancer cells [72].

The downregulation of PDCD4 was one of the critical factors determining the chemoresistant capabilities of BC cells. Our results demonstrated that Dox-induced chemoresistance was linked to the downregulation of PDCD4 in variant B cells, which promotes the invasion and metastasis of tumor cells. A recent study characterized the dynamic relationship between PDCD4 and EMT-associated proteins, and the suppression of PDCD4 was correlated with increased cell invasion, a key cancer hallmark [73]. Additionally, the deletion of PDCD4 contributed to the development of chemoresistance in models of glioblastoma multiforme [54]. These findings are consistent with the results of our TNBC model. Specifically, PDCD4 could regulate the expression of Sin1, p53, E-cadherin, CMyb, and AMyb, which are targets involved in controlling neoplastic processes such as motility, invasion, and cell growth [42,43,44]. These observations could explain the results of our TNBC model experiments. Moreover, our results indicated that PDCD4 was mainly localized in the cytoplasm, and the subtle regulation of the interactions of eIF4A suggests that this process was highly regulated even at high Dox concentrations. Similarly, a reduction in the levels of nuclear PDCD4 in lung carcinoma cells was linked to tumor progression [56], which was consistent with our findings.

Ubiquitination and proteasome degradation of PDCD4 through E3 ubiquitin ligase has been described in ovarian and endometrial cancer cells [16]. Likewise, HER2 activation could promote the loss of PDCD4 by activating MAPK, AKT, and miR-21 in aromatase inhibitor-resistant BC cells [55]. Therefore, these mechanisms could contribute to sustaining the expression of PDCD4. Our research group is currently characterizing the impact of the mTOR/p70S6K signaling pathway on PDCD4 expression. 

There is a potential association between cancer hallmarks (e.g., migration and invasion), ER stress and the UPR, and this connection could be mediated by chaperones such as BiP and the triggering of transcription factors such as Nrf2 and XBP1s. Here, we demonstrated that the chaperone BiP was localized in the extracellular media. Furthermore, our data suggest the role of regulation on FAK activation and MMP-9 secretion; moreover, these phenomena could be driven by high LDL concentrations. Likewise, we evaluated the regulatory role of Cry using Sal and Tum as references.

In this regard, the potential role of eIF4A is the novel data. In this regard, the significance of the eIF4A approach is supported by the pro-oncogenic and pro-survival mRNAs dependent on eIF4A processing [74]. For instance, the activation of CXCR4 signaling, one of the most significant chemokine receptors involved in BC metastasis, increased the reliance on eIF4A translation of the oncoproteins ROCK1, survivin, Mdm2, and cyclin D1 [75]. Critically, eIF4A could mediate EMT, which is associated with tumor cell invasion and metastasis [76]. Aberrant activation of FAK signal transduction in tumor cells is correlated with their invasion ability and potential effects on metastasis. Our results suggested that eIF4A affects the expression and activation of FAK, as demonstrated by siRNA knockdown and overexpression experiments in the TNBC variants (Figure 7). This association suggests that FAK expression depends on the activation of eIF4A. In turn, this phenomenon could be mediated by the PI3K/Akt/mTOR signaling cascade. These data are highly relevant, as metastasis remains the primary cause of morbidity and mortality in cancer patients [77]. Moreover, ApoB-lipoproteins (VLDL, IDL, and LDL) and their components modulate intracellular metabolism and have been associated with the development of neoplastic phenomena such as proliferation, anchorage-independent growth, epithelial-mesenchymal transition, and cancer invasion [34].

In gastric tumor cells, FAK-silencing accentuated the effect of 5-fluorouracil, which was associated with the activation of caspase-3 activity [78]. Gemcitabine-resistant pancreatic cancer cells have been reported to exhibit high activation of p-FAK (Tyr^397^), and inhibitory RNAi treatment increases gemcitabine-induced cytotoxicity [79]. In our conditions, concomitant Dox/Cry and Dox/Tum treatments synergistically inhibited p-FAK^397^. Particularly, Dox/Cry treatment exhibited the strongest inhibitory effects on the FAK expression of both parental and variant B cells. Co-treatment with Cry (25 µM) and low Dox concentrations (250–500 nM) thus constitutes a promising sensitizing treatment for regulating FAK signaling. Therefore, FAK-regulated mechanisms impact the sensitivity of anti-tumoral treatments. This highlights the relevance of our data, as cancer relapse after chemotherapy remains one of the leading causes of death [80]. Importantly, our study was the first to describe the dependence of FAK on eIF4A.

eIF4A overexpression induced malignant progression in an acute lymphocytic leukemia model [81]. Moreover, given that the Sal treatments rendered unpromising results, we explored alternatives to sensitize cancer cells. Several small molecules known to disrupt eIF4A RNA helicase have been reported to possess anticancer activity both in vitro and in vivo, including rocaglates, hippuristanol, rohinitib, and pateamine A (PatA) [82,83]. PatA, a macrolide marine compound, could slightly inhibit cap-dependent translation in chronic lymphocytic leukemia cells [84], thus highlighting the promising therapeutic potential of molecules derived from natural products. In our study, the novel combination of Cry and Dox exhibited synergistic anticancer activity against TNBC cells, re-establishing the sensitivity of Dox-resistant cells and impairing the viability of parental cells. This suggests the importance of pharmacologic eIF4A inhibition mediated by Cry. Moreover, selective compounds inhibit the translation of oncogenic gene drivers by increasing the affinity between eIF4A and specific polypurine sequence motifs in the 5′UTR [17].

The chemosensitization of Cry and the effect on FAK regulation have important implications in crucial processes such as migration and invasion, both of which are associated with metastasis. Previous studies have suggested that the chemoresistance phenotype could modify the migratory and invasion capacities of cancer cells. In this regard, FAK is a non-receptor protein kinase involved in tumor migration, adhesion, invasion, and metastasis [85]. Therefore, FAK Tyr^397^ phosphorylation maximizes its catalytic activity, thus inducing the phosphorylation of several downstream targets [86], which could be modulated by eIF4A.

Our group previously characterized the role of LDL hypercholesterolemia on the process of epithelial-mesenchymal transition and its connection with cancer hallmarks such as metastasis and chemoresistance [34]. LDL endocytosis and the induction of invasion capability dependent on LDL were observed in both variants. We have previously discussed the role of targets that regulate cholesterol metabolism, including de novo synthesis, endocytosis, and oxidation, which contribute to cancer development. Specifically, we discussed the mechanisms associated with sterol regulatory element-binding protein 2 (SREBP-2)/mevalonate, as well as metabolites derived from cholesterol oxidation, such as oxysterols and epoxy-cholesterols [34]. 

According to our findings, high levels of pro-atherogenic LDL could promote invasiveness and chemoresistance, which we have characterized in other subtypes. Previous studies have also proposed that metabolic alterations play a central role in the chemoresistance of cancer cells [34]. Therefore, LDL dyslipidemia is a critical condition to consider during the establishment of regime treatments and its efficacy evolution.

Cry is one of the primary active constituents isolated from the root of *Salvia brandegeei*, and our findings demonstrated that Cry + Dox co-exposure could effectively sensitize TNBC cell variants, even more, when eIF4A was overexpressed in variant B cells. Our data confirmed the interaction between Cry and eIF4A through fluorescence characterization. Additionally, our molecular docking analyses suggested that this interaction likely interferes with the RNA binding domain. These findings have critical implications for the expression of oncogenic drivers. Therefore, our data suggest that the specific disruption of protein synthesis is a promising therapeutic strategy for the treatment of aggressive cancers such as TNBC.

## 5. Conclusions

Our results suggest that the UPR is an adaptive mechanism that is crucial for the acquisition of chemoresistance. Therefore, the PDCD4/eIF4A/FAK signaling pathway constitutes a crucial basis for the discovery and characterization of novel cancer drug targets. Moreover, new therapeutic strategies could be developed based on the structure of cryptotanshinone derived from natural sources. Metabolic factors such as LDL dyslipidemia could also potentiate the development of chemoresistance and impact the efficacy of anti-cancer treatments. More importantly, the chemoresistance of variant B in our study confirmed that novel therapeutic approaches can be developed based on the regulation of protein translation initiation factors.

## Data Availability

The data presented in this study are available in article and Supplementary Materials.

## Supplementary Materials

The following supporting information can be downloaded at: https://www.mdpi.com/article/10.3390/cells11244069/s1, Supplementary Figure S1. Behavior of sensitive variant F. Supplementary Figure S2. Localization of BiP in extracellular media of parental and variant B cells. Supplementary Figure S3. Localization of XBP1s in extracellular media of parental and variant B cells. Supplementary Figure S4. Phenotypic characterization of parental and variant B cells under concomitant Cry/Dox and Tum/Dox.
