# Supplementary material for: eIF4A/PDCD4 Pathway, a Factor for Doxorubicin Chemoresistance in a Triple-Negative Breast Cancer Cell Model
Source: Cells. 2022 Dec 15;11(24):4069. doi: 10.3390/cells11244069 (PMC9776898; doi:10.3390/cells11244069)

eIF4A/PDCD4 pathway, a factor for doxorubicin chemoresistance in a triple-negative breast cancer cell model

Alina González-Ortiz[1,2&], Angel Pulido-Capiz[1,3&], César Y. Castañeda-Sánchez[1,2], Esmeralda Ibarra-López[1,2] , Octavio Galindo-Hernández[1,2], Maritza Anahí Calderón-Fernández[1,2], Leslie Y. López-Cossio[1,2], Raúl Díaz-Molina[1,2], Brenda Chimal-Vega[1,2], Nicolás Serafín-Higuera[4], Iván Córdova-Guerrero[5], Victor García-González[1,2*]

[1] Departamento de Bioquímica, Facultad de Medicina Mexicali, Universidad Autónoma de Baja California, Mexicali 21000, Baja California, México.

[2] Laboratorio Multidisciplinario de Estudios Metabólicos y Cáncer, Universidad Autónoma de Baja California, Mexicali 21000, Baja California, México.

[3] Laboratorio de Biología Molecular, Facultad de Medicina Mexicali, Universidad Autónoma de Baja California, Mexicali 21000, Baja California, México.

[4] Facultad de Odontología Mexicali, Universidad Autónoma de Baja California, Mexicali 21000, Baja California, México.

[5] Facultad de Ciencias Químicas e Ingeniería, Universidad Autónoma de Baja California, Tijuana, México.

[&]Equal contribution

*Corresponding author

Supplementary figures

Sup. Fig. S1. Behavior of sensitive variant F. **A**) MTT assay in parental cells and variant F under increasing doxorubicin (Dox) concentrations, mean values are presented (n = 6, mean ± SD), *p< 0.001, **p< 0.05, respect to parental cells. **B**) XBP1s expression under salubrinal (Sal) and Dox treatments. β-actin was used as a loading control. **C**) Analysis of Dox treatment (500 nM) through flow cytometry.

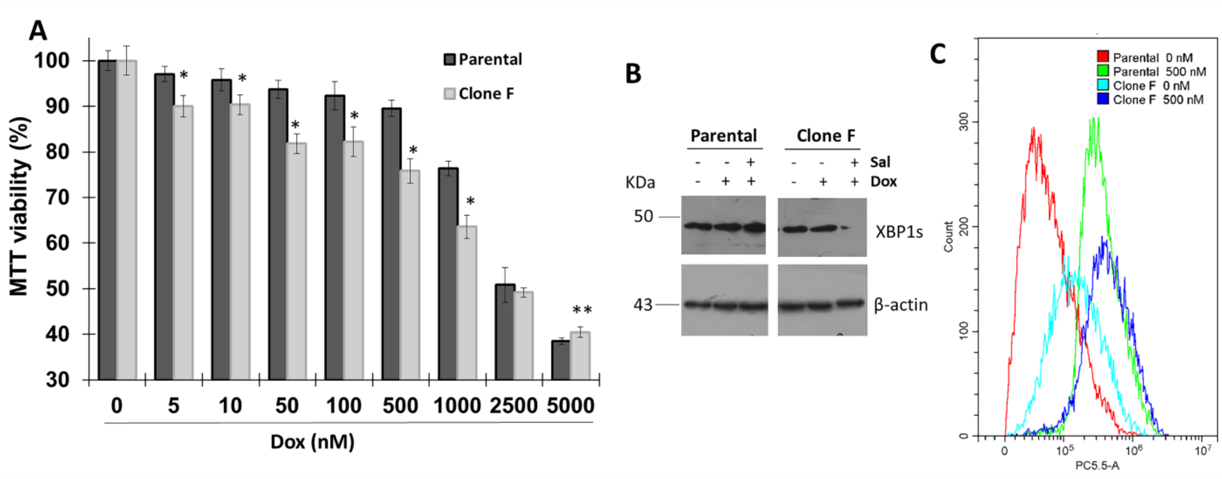

Sup. Fig. S2. Localization of BiP in extracellular media of parental and variant B cells. Identification by western-blot of BiP in 10X concentrated supernatant media of parental and variant B cells under the Doxorubicin (Dox) treatment (100 nM) and concomitant Salubrinal (Sal) (25 µM), Cryptotanshinone (Cry) (25 µM), and Tunicamycin (Tum) (1 µg/mL). Coomassie stain on PVDF membranes and β-actin were used as loading controls.

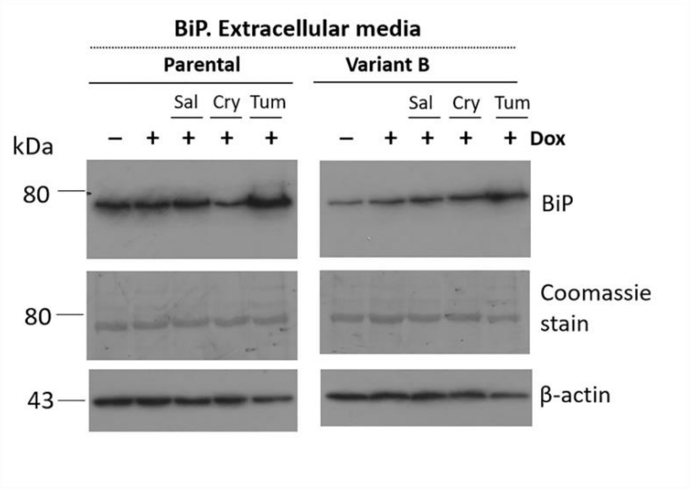

Sup. Fig. S3. Localization of XBP1s in extracellular media of parental and variant B cells. Identification by western blot of XBP1s in 10X concentrated supernatant media of parental and variant B cells under LDL (25 µg/mL) and Doxorubicin (Dox) (100 nM) treatment. Coomassie stain was used as a loading control.

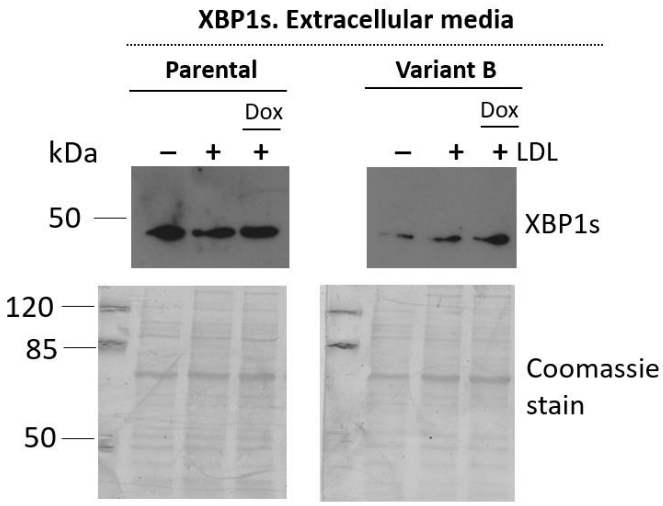

Sup Fig. S4. Phenotypic characterization of parental and variant B cells under concomitant Cry/ Dox and Tum/Dox. **A**) Effect of concomitant Dox (100 nM) treatment with Cryptotanshinone (Cry) (25 µM) and Tunicamycin (Tum) (1 µg/mL) by quantification of MMP-9 collagen degradation. Results are expressed as a percent of control. Employing the same conditions, representative images of optical microscopy. In parental cells, a representative image corresponding to control (**B**), Cry plus Dox treatment (**C**), and Tum plus Dox treatment (**D**). The same assays showed in variant B, control (**E**), Cry plus Dox treatment (**F**), and Tum plus Dox treatment (**G**).

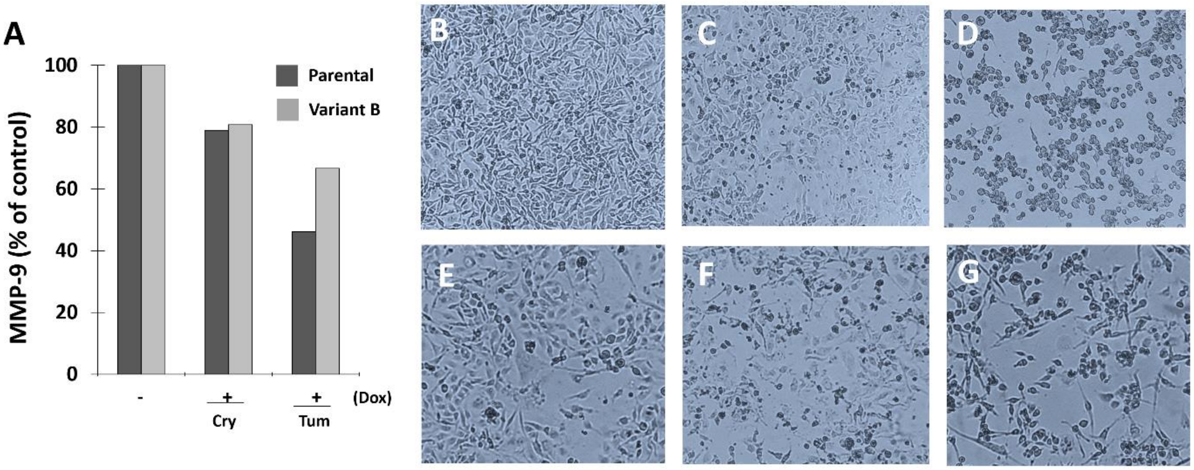

Supplement: Supplementary file 1 [file cells-11-04069-s001.zip › cells-1862462-supplementary.pdf]
